# Comprehensive subtyping of Parkinson’s disease patients with similarity fusion: a case study with BioFIND data

**DOI:** 10.1038/s41531-021-00228-0

**Published:** 2021-09-17

**Authors:** Matthew Brendel, Chang Su, Yu Hou, Claire Henchcliffe, Fei Wang

**Affiliations:** 1 Institute for Computational Biomedicine, Department of Physiology and Biophysics, Weill Cornell Medicine of Cornell University, New York, NY 10065 USA; 2 Department of Health Service Administration and Policy, Temple University, Philadelphia, PA 19122 USA; 3 Department of Population Health Sciences, Weill Cornell Medical College, New York, NY 10065 USA; 4 Department of Neurology, University of California Irvine, Irvine, CA 92697 USA

**Keywords:** Parkinson's disease, Parkinson's disease

## Abstract

Parkinson’s disease (PD) is a complex neurodegenerative disorder with diverse clinical manifestations. To better understand this disease, research has been done to categorize, or subtype, patients, using an array of criteria derived from clinical assessments and biospecimen analyses. In this study, using data from the BioFIND cohort, we aimed at identifying subtypes of moderate-to-advanced PD via comprehensively considering motor and non-motor manifestations. A total of 103 patients were included for analysis. Through the use of a patient-wise similarity matrix fusion technique and hierarchical agglomerative clustering analysis, three unique subtypes emerged from the clustering results. Subtype I, comprised of 60 patients (~58.3%), was characterized by mild symptoms, both motor and non-motor. Subtype II, comprised of 20 (~19.4%) patients, was characterized by an intermediate severity, with a high tremor score and mild non-motor symptoms. Subtype III, comprised of 23 (~22.3%) patients, was characterized by more severe motor and non-motor symptoms. These subtypes show statistically significant differences when looking at motor (*on* and *off* medication) clinical features and non-motor clinical features, while there was no clear difference in demographics, biomarker levels, and genetic risk scores.

## Introduction

Parkinson’s disease (PD) is a complex and progressively debilitating neurodegenerative disease with distinct clinical manifestations of motor and non-motor characteristics^1^. Despite its common core characteristics, it is heterogeneous in terms of clinical manifestations, progression course, treatment response, genetic underpinnings, and biomarker readouts and pathology. Identification of PD subtypes may therefore lead to further insights into mechanisms of disease, potential therapeutic targets, and could dramatically enhance patient care and clinical trial design^2–4^. The rationale of this study is to comprehensively integrate heterogeneous data provided by the BioFIND observational study, a cohort of patients with “typical” PD at moderate-to-advanced stages, in order to identify meaningful subtypes of PD patients^5^.

Conventionally, PD subtypes have been identified empirically based on clinical observations and analyses of cohort studies, by focusing on informative motor and non-motor variables^6–8^. For example, PD can be divided into tremor-dominant (TD), and postural instability and gait difficulty (PIGD) subtypes based on motor manifestations as measured by the Movement Disorder Society Unified PD rating scale (MDS-UPDRS)^6^, and such standardized rating scales may also be used to classify patients according to rates of progression. PD subclassification may also be based on associated non-motor manifestations, for example, REM sleep behavior disorder (RBD)^9^. There have been recent efforts to develop treatment based upon genetic subtypes, for example, GBA or LRRK2-associated PD^10^. However, these conventional subtypes methods only characterize patients based on a very limited aspect of this complex disease. More comprehensive approaches that can consider multiple different aspects of patient characteristics are needed.

In this context, researchers have turned to data-driven perspectives, where patient subtyping is transformed into a typical clustering problem^11–16^. These works have focused on several different types of data collected from patients besides just clinical assessments, which include neuroimaging data^17–19^, genomic data^20^, and neurophysiological assessment data^21^. Without any prior assumption patients are grouped into clusters, each of which corresponds to a specific subtype, such that patients within the same subtype manifest similar PD characteristics while those from different subtypes are distinct. Generally, the existing data-driven PD subtyping approaches represented each patient by an array of all or some of his/her features (e.g., motor and non-motor assessments), which is then directly fed into the standard clustering models, e.g., k-means and hierarchical agglomerative clustering^22^ to derive the subtypes. However, a typical limitation of these methods is that, by directly aggregating heterogeneous data as input, they are not able to capture the underlying patterns and interactions within a specific type of data (such as MDS-UPDRS I, REM Behavioral Disorder (RBD) score and Montreal Cognitive Assessment score, all non-motor data) and that between different types of data (such as non-motor and motor data). Previous methods that have aimed to tackle this problem have used similarity matrix fusion approaches^23,24^.

In this paper, we propose a data-driven subtyping method which integrates both motor and non-motor characteristics of PD patients. More precisely, we first calculated two similarity matrices in terms of motor and non-motor manifestations, which indicate the pairwise similarities between all patients with respect to each set of features. Then we integrated the two similarity matrices through an optimization based fusion method, which generates a final pairwise patient similarity measure that can be used for further analyses. Third, hierarchical agglomerative clustering was performed over the integrated similarity matrix to identify the comprehensive subtypes of PD patients. Finally, we evaluated differences in demographics, clinical characteristics (motor and non-motor), biomarkers and genetic information (genetic risk score) between subtypes/clusters by statistical testing.

## Results

### Patients and baseline description

Of the 118 PD participants in the BioFIND study, 103 patients had complete clinical data and were included in this study, consisting of 63 (61.2%) males and 40 (38.8%) females with an average age of 71.87 ± 6.44. The participants’ years of education were 16.87 ± 2.95. A family history of PD (either grandparents, parents, siblings, or children with a diagnosis of PD) was reported in 19 (18.4%) participants. The mean MDS-UPDRS I and II and the MDS-UPDRS III on and off medication scores were 9.65 ± 5.82, 11.24 ± 6.59, 28.43 ± 14.22, and 38.59 ± 13.21, respectively.

### PD subtypes

Through jointly tuning cluster number and MBBS hyperparameters, the Silhouette index suggested three distinct clusters: Subtype I, comprised of 60 patients (58.3%); Subtype II, comprised of 20 (19.4%) patients; and Subtype III, comprised of 23 (~22.3%) patients. Through cluster stability analysis, we show that these clusters represent distinct patient populations (Supplementary Fig. 1). We also see that the most incorrect clustering during subset analysis is true subtype II clusters being clustered as Subtype I. In addition, by adding an additional biomarker matrix to the optimization process, the clusters stay relatively the same, with a rand index of 0.9052. Interestingly, we see that the coefficient for the added biomarker matrix had the lowest coefficient, showing that it had the lowest contribution to the clustering results.

### Subtype Characteristics

**Fig. 1:**
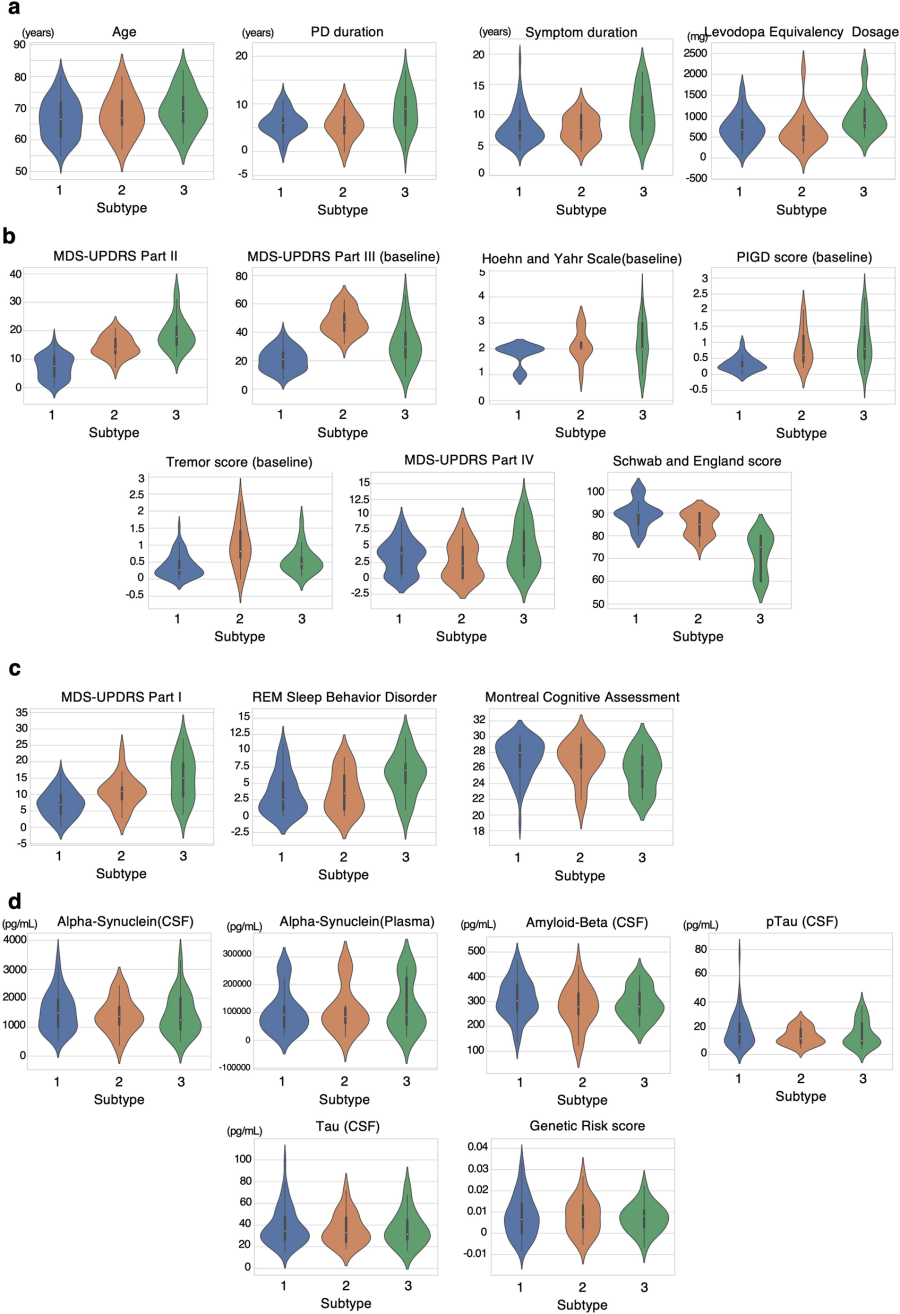
Violin plots showing characteristics of the identified subtypes. Feature distributions of (**a**) demographics, (**b**) motor clinical assessments, (**c**) non-motor clinical assessments, and (**d**) biospecimen measurements/genetic risk score. The violin plots show a kernel density estimate of the feature distribution. The white dot within each plot represents the median, the edges of the box represent the interquartile and the line represents 1.5 times the interquartile range.

Statistical testing was used to determine how the subtype populations differed and violin plots were used to visualize the data distributions per clusters (Table 1 and Fig. 1). For this section, a difference in values will refer to a statistically significant difference based on the statistical methods discussed in the previous section.

**Table 1:** Characteristics of the identified PD subtype.

Variable	Subtype I (*n* = 60)	Subtype II (*n* = 20)	Subtype III (*n* = 23)	P-value	FDR-corrected P-value	P-value adjusting for age and PD duration	Post-Hoc
*Demographics*							
Age, mean (SD), year	66.733 (6.241)	67.9 (6.406)	69.522 (6.251)	0.193	0.3228	NA	–
Family history, No. (%)	11 (18.3)	3 (15)	5 (21.7)	0.883	0.9123	NA	–
Education years, mean (SD)	17.2 (2.922)	16.8 (3.238)	16.087 (2.745)	0.307	0.4408	0.436	–
Gender (male), No. (%)	35 (58.3)	14 (70.0)	14 (60.9)	0.724	0.8109	NA	–
Symptom duration, mean (SD), year	7.833 (2.799)	7.75 (2.173)	10.391 (3.775)	0.009	0.0265	NA	III vs rest
PD duration, mean (SD), year	5.967 (2.604)	5.55 (2.929)	8.478 (4.241)	0.024	0.0498	NA	III vs rest
Levodopa equivalency dosage, mean (SD), mg	705.695 (360.549)	634.25 (421.231)	986.260 (426.057)	0.005	0.0175	0.08	III vs rest
Ethnicity (caucasian), No. (%)	55 (91.7)	19 (95)	23 (100)	0.443	0.5290	NA	–
*Other CNS diseases*							
Epilepsy, no. (%)	0 (0.0)	0 (0.0)	0 (0.0)	–	–	NA	–
Head trauma, No. (%)	7 (11.7)	0 (0.0)	3 (13)	0.301	0.4408	NA	–
Narcolepsy, no. (%)	0 (0.0)	0 (0.0)	1 (4.3)	0.417	0.5189	NA	–
Depression, no. (%)	16 (26.7)	4 (20)	13 (56.5)	0.02	0.0467	NA	III vs rest
Stroke, no. (%)	1 (1.7)	0 (0.0)	0 (0.0)	1	1.0000	NA	–
Restless leg Syndrome, no. (%)	6 (10)	2 (10)	8 (34.8)	0.024	0.0498	NA	–
Parkinsonism, no. (%)	51 (85)	18 (90)	22 (95.7)	0.444	0.5290	NA	–
*Non-motor manifestations*							
UPDRS I anxious mood, mean (SD)	0.383 (0.64)	0.65 (0.745)	0.957 (0.928)	0.024	0.0498	NA	I vs III
UPDRS I apathy, mean (SD)	0.217 (0.454)	0.25 (0.55)	0.957 (1.065)	0.002	0.0093	NA	I vs III
UPDRS I constipation, mean (SD)	0.567 (0.647)	1.05 (0.945)	1.13 (0.869)	0.007	0.0218	NA	I vs III
UPDRS I cognitive impairment, mean (SD)	0.467 (0.623)	0.55 (0.686)	1.174 (0.984)	0.014	0.0373	NA	I vs III
UPDRS I dopamine dysregulation syndrome, mean (SD)	0.067 (0.252)	0.15 (0.489)	0.304 (0.822)	0.069	0.1246	NA	–
UPDRS I depressed moods, mean (SD)	0.283 (0.555)	0.65 (0.813)	0.87 (1.058)	0.016	0.0390	NA	I vs III
UPDRS I fatigue, mean (SD)	0.783 (0.715)	1.15 (1.089)	1.652 (1.191)	0.003	0.0112	NA	I vs III
UPDRS I Hallucinations and psychosis, mean (SD)	0.1 (0.354)	0.1 (0.308)	0.261 (0.541)	0.402	0.5116	NA	–
UPDRS I Lightheadedness on standing, mean (SD)	0.45 (0.723)	0.65 (0.988)	0.87 (1.014)	0.01	0.0280	NA	I vs III
UPDRS I pain and other sensations, mean (SD)	0.85 (0.88)	1.25 (1.02)	1.826 (1.114)	0.001	0.0051	NA	I vs rest
UPDRS I daytime sleepiness, mean (SD)	1.1 (0.838)	1.35 (0.813)	1.609 (0.891)	0.316	0.4424	NA	–
UPDRS I total score, mean (SD)	7.25 (4.049)	10.7 (4.769)	15.0 (6.822)	<0.0001	<0.0001	NA	All Comparisons
RBD questionnaire score, mean (SD)	3.45 (3.127)	3.75 (3.143)	6.348 (3.284)	0.003	0.0112	NA	III vs rest
Montreal cognitive assessment score, mean (SD)	27.283 (2.373)	26.9 (2.532)	25.565 (2.519)	0.016	0.0390	NA	I vs III
RBD subtype (positive), No. (%)	21 (35)	7 (35)	18 (78.3)	0.001	0.0051	NA	III vs rest
*Motor manifestations*							
schwab and england assessment of daily living, mean (SD)	89.667 (5.813)	85.0 (5.13)	71.957 (8.757)	<0.0001	<0.0001	NA	All Comparisons
Hoehn and Yahr score on medication, mean (SD)	1.783 (0.415)	2.15 (0.587)	2.174 (0.834)	<0.0001	0.0002	NA	I vs rest
Hoehn and Yahr score off medication, mean (SD)	1.967 (0.45)	2.25 (0.786)	2.565 (0.728)	0.003	0.0112	NA	I vs III
UPDRS II score, mean (SD)	7.267 (4.129)	14.45 (3.62)	18.826 (5.646)	<0.0001	<0.0001	<0.0001	All Comparisons
UPDRS III score on medication, mean (SD)	20.983 (7.961)	47.7 (8.998)	31.087 (14.177)	<0.0001	<0.0001	NA	All Comparisons
UPDRS III score off medication, mean (SD)	34.0 (11.423)	48.1 (11.742)	42.304 (13.636)	<0.0001	0.0002	<0.0001	I vs rest
UPDRS IV functional impact of dyskinesias, mean (SD)	0.15 (0.444)	0.05 (0.224)	0.217 (0.422)	0.361	0.4931	NA	–
UPDRS IV painful OFF-state dystonia, mean (SD)	0.467 (0.769)	0.45 (0.759)	0.739 (1.054)	0.397	0.5116	NA	–
UPDRS IV functional impact of fluctuations, mean (SD)	0.717 (0.804)	0.65 (0.933)	1.174 (1.302)	0.096	0.1680	NA	–
UPDRS IV complexity of motor fluctuations, mean (SD)	0.867 (0.812)	0.5 (0.607)	1.174 (1.114)	0.254	0.3951	NA	–
Time spent in the OFF state, mean (SD)	0.767 (0.593)	0.8 (1.105)	0.87 (0.694)	0.044	0.0880	NA	–
UPDRS IV time spent with dyskinesias, mean (SD)	0.45 (0.622)	0.35 (0.489)	0.565 (0.59)	0.779	0.8554	NA	–
UPDRS IV total score	3.517 (2.593)	2.8 (2.876)	4.739 (3.596)	0.196	0.3228	NA	–
PIGD score on medication, mean (SD)	0.32 (0.233)	0.84 (0.56)	1.035 (0.684)	<0.0001	<0.0001	NA	I vs rest
PIGD score off medication, mean (SD)	0.48 (0.355)	0.9 (0.61)	1.252 (0.789)	<0.0001	<0.0001	NA	All Comparisons
Tremor Score on medication, mean (SD)	0.4 (0.338)	0.995 (0.625)	0.557 (0.389)	0.0004	0.0025	NA	II vs rest
Tremor score off medication, mean (SD)	0.624 (0.413)	0.986 (0.573)	0.874 (0.525)	0.006	0.0198	0.006	I vs II
Motor subtype on medication: TD, No. (%)	30 (50)	11 (55)	4 (17.39)	0.054	0.1008	NA	–
Motor subtype on medication: Indeterminate, No. (%)	9 (15)	3 (15)	5 (21.74)				
Motor subtype on medication: PIGD, No. (%)	21 (35)	6 (30)	14 (60.87)				
Motor subtype off medication: TD, No. (%)	33 (55)	13 (65)	8 (34.78)	0.286	0.4329	NA	–
Motor subtype off medication: Indeterminate, No. (%)	8 (13.33)	1 (5)	3 (13.04)				
Motor subtype off medication: PIGD, No. (%)	19 (31.67)	6 (30)	12 (52.17)				
*Biospecimen*							
Amyloid-beta CSF, mean (SD), pg/mL	311.292 (76.653)	280.345 (80.665)	292.508 (62.919)	0.232	0.3712	0.29	–
Alpha-synuclein CSF, mean (SD), pg/mL	1537.017 (670.56)	1406.865 (576.933)	1419.97 (687.962)	0.645	0.7371	0.395	–
Alpha-synuclein Plasma, mean (SD), pg/mL	106193.611 (77202.14)	112732.203 (82070.173)	123828.093 (91534.726)	0.821	0.8717	NA	–
pTau CSF, mean (SD), pg/mL	18.264 (12.669)	13.992 (6.397)	15.673 (9.831)	0.556	0.6487	NA	–
Tau CSF, mean (SD), pg/mL	38.283 (16.985)	36.143 (14.334)	36.383 (15.826)	0.825	0.8717	0.549	–
*Genetic factor*							
Genetic risk score, mean (SD)	0.008 (0.01)	0.008 (0.008)	0.007 (0.007)	0.896	0.9123	0.99	–

Abbreviations: SD: Standard Deviation, PD: Parkinson Disease, CNS: Central Nervous System, UPDRS: Unified Parkinson Disease Rating Scale, RBD: REM Sleep Behavioral Disorder, PIGD: Postural Instability and Gait Disturbance, TD: Tremor Dominant, CSF: Cerebrospinal Fluid

For continuous variables, analysis of variance (ANOVA) with Tukey’s HSD post-hoc analysis (normal distribution) or Kruskal–Wallis test with Wilcoxon rank sum test with Benjamini–Hochberg correction (non-normal distribution) were performed. Fisher’s Exact Tests were performed for categorical data, with Benjamini–Hochberg post-hoc analysis. The nominal p-values were also corrected using Bejamini–Hochberg FDR correction. Analysis of covariance (ANCOVA) was performed adjusting age and disease duration at baseline. NA represents measurement not taken.

There were no significant differences in mean age, sex, years of education, nor ethnicity between the three subgroups. We note, however, that there is very little diversity in ethnicity for this PD cohort.

PD duration (time since diagnosis) and symptom duration were both significantly longer in Subtype III than in Subgroups I and II (8.478 ± 4.241, 10.391 ± 3.775, p = 0.024, 0.009 for Subtype III, compared to 5.967 ± 2.604, 7.833 ± 2.799 and 5.55 ± 2.929, 7.75 ± 2.173 for I and II, respectively). Study participants in Subtype III were also treated with a significantly greater anti-PD medication dose, measured as LEDD (986.3 ± 426.1, *p* = 0.005) when compared to the Subtypes I and II (705.695 ± 360.549 and 634.25 ± 421.231 for Subtype I and II, respectively).

There was a significant difference between subtypes for the MDS-UPDRS II score with Subtype I least affected, Subtype III most affected, and Subtype II intermediate (7.267 ± 4.129, 14.45 ± 3.62, 18.826 ± 5.646 for Subtype I, II, and III, *p* < 0.0001). This corresponds to a difference in Schwab and England Assessment of Daily Living scores, also a self-reported assessment (*p* = < 0.0001). There were also significant differences between subtypes in the MDS-UPDRS III scores both on and off medication with Subtype I least affected, but with Subtype II more affected than Subtype III (20.983 ± 7.961, 47.7 ± 8.998, 31.087 ± 14.177, *p* < 0.0001 and 34.0 ± 11.423, 48.1 ± 11.742, and 42.304 ± 13.636, *p* < 0.0001). There was no significant difference between the subtypes for overall MDS-UPDRS IV scores, although numerically Subtype III has the most severe score, Subtype II the mildest, and Subtype I is intermediate (3.517 ± 2.593, 2.8 ± 2.876, 4.739 ± 3.596, *p* = 0.196 for Subtype I, II, and III respectively). The Hoehn and Yahr stage for Subtype II also has the lowest difference of the three subtypes between the on and off medication states (2.15 ± 0.587 off vs. 2.25 ± 0.786 on). Of note, there is a higher Tremor score for subtype II (0.995 ± 0.625), and this does not change between “on” and “off” states in this Subtype, in contrast to Subtypes I and II (0.4 ± 0.338 and 0.557 ± 0.389 for Subtype I and II respectively).

We analyzed differences between phenotypic subtypes tremor dominant (TD), postural instability-gait disturbance (PIGD) and indeterminate between subgroups in the on and off states. Based on previous literature, individuals may shift from one phenotypic subtype to another between the *on* and *off* states^7^. If a patient goes from a TD classification while off medication, to another classification while on medication, the patient is considered a “TD-shifter”, and similarly for PIGD. Patients who were indeterminate in the *off* state, were grouped into a separate category. Supplementary Fig. 3 demonstrates that TD patients in Subgroup III are more likely to shift to another phenotypic subtype in response to medication, in contrast to Subgroups I and particularly II, in which the majority of TD individuals do not shift in response to medication.

A number of differences in non-motor features were also evident between the subtypes. For the overall MDS-UPDRS Part I score as well as 8/13 individual questions (cognition, anxiety, apathy, depression, fatigue, pain, lightheadedness on standing, constipation), there was a significantly worse score in Subtype III compared with Subtype I, with subtype II having an intermediate value (7.25 ± 4.049, 10.7 ± 4.769, and 15.0 ± 6.822, *p* < 0.0001 for Subtype I, II, and III). Those in Subtype III were more likely to have clinically diagnosed depression (56.5%, *p* = 0.02) than those in Subtypes I and II (26.7% and 20% respectively). Differences in individual scores for individual questions on hallucinations and psychosis, night time sleep problems, daytime sleepiness, and the presence of dopamine dysregulation syndrome did not meet significance, and were of borderline statistical significance for urinary problems, although we note that as for the other non-motor items above, scores for each of these features were numerically best for Subtype I, worst for Subtype III and typically intermediate for Subtype II. Independent of the MDS-UPDRS measures, mean score from the RBD questionnaire was significantly higher (more affected) in Subtype III compared with Subtypes I and II (3.45 ± 3.127, 3.75 ± 3.143, and 6.348 ± 3.284, *p* = 0.003 for Subtype I, II, and III). Subtype I and II have a 35% RBD-positive subtype, whereas Subtype III had a much higher proportion of patients with the RBD-positive subtype (78.3%). Cognition was also more affected in Subtype III compared with Subtypes I and II as rated by Montreal Cognitive Assessment Scores (27.283 ± 2.373, 26.9 ± 2.532, and 25.565 ± 2.519, *p* = 0.016 for Subtype I, II, and III).

### Biomarkers and genetic factors

No differences were observed in both classic biomarker measurements (α-Synuclein, Aβ, and Tau) and genetic risk score. However, when analyzing a more exploratory set of biomarkers from taken from mass-spectrometry data from our patient cohort, we did identify a set of protein biomarkers that are significantly different in subtype III, compared to subtype I and II. Of note, we see a significant increase in levels of the proteins for AMBP, APOA4, C9, F11, FGA, LYZ, MBL2, PLD4, and SERPINA10, and the protein that was lower was FTL (Table 2 and Supplementary Fig. 2).

**Table 2:** List of statistically significant proteins between clusters (Numerical values are relative quantification of each protein in CSF).

Protein	Subtype I (*n* = 60)	Subtype II (*n* = 20)	Subtype III (n = 23)	ANOVA P-value	FDR-corrected P-value	Post-Hoc (Tukey-HSD)
AMBP, mean (SD)	1.1038 (0.3206)	1.1635 (0.3499)	1.4268 (0.4255)	0.0013	0.0439	III vs rest
APOA4, mean (SD)	1.0654 (0.3505)	1.0794 (0.2420)	1.3963 (0.5126)	0.0017	0.0491	III vs rest
C9, mean (SD)	1.2819 (0.4093)	1.2747 (0.4344)	1.7141 (0.6859)	0.0014	0.0439	III vs rest
F11, mean (SD)	1.0155 (0.3206)	0.9608 (0.2000)	1.3286 (0.4481)	0.0003	0.0288	III vs rest
FGA, mean (SD)	1.1921 (0.4835)	0.9537 (0.3375)	1.5521 (0.6936)	0.0009	0.0439	III vs rest
FTL, mean (SD)	1.1146 (0.2791)	1.2598 (0.4105)	0.9328 (0.1245)	0.0012	0.0439	III vs rest
LYZ, mean (SD)	1.0037 (0.2379(	1.0666 (0.2040)	1.3162 (0.4367)	0.0001	0.0168	III vs rest
MBL2, mean (SD)	0.8587 (0.4244)	0.8595 (0.2951)	1.5076 (0.7946)	<0.00001	0.0009	III vs rest
PLD4, mean (SD)	1.0010 (0.2070)	1.1444 (0.2176)	1.3071 (0.5469)	0.0057	0.0370	I vs III
SERPINA1, mean (SD)	1.1309 (0.3593)	1.1220 (0.2959)	1.4475 (0.4428)	0.0226	0.0491	III vs rest

## Discussion

In this study, we developed a data-driven subtyping method for moderate-to-advanced PD, via comprehensively considering the motor and non-motor manifestations in a cross-sectional cohort. Different from the existing data-driven subtyping studies^11^ which concatenated motor and non-motor variables to derive clusters, we calculated patient-wise similarity matrices based on motor and non-motor manifestations separately and, using our MBBS algorithm^25^, produced a comprehensive, integrated algorithm via improving the cluster structures. The weights of the motor and non-motor matrices to construct the integrated similarity matrix are 0.6994 and 0.3006, respectively, indicating motor symptoms have a major contribution to defining the PD subtypes we have determined in this study. This study can be applicable to many other diseases, as well as different modes of data collected from patients. The MBBS algorithm can include more than 3 datasets for fusion, so it may be of interest in future studies to combine neuroimaging and genomic data, in addition to clinical data, to analyze PD subpopulations.

Through the use of the data-driven method, we have identified three PD subtypes within the BioFIND cohort. Significant differences in motor and non-motor signs and symptoms were observed among the three subtypes, in addition to differences in medication response. Specifically, we can interpret the three subtypes as follows. Subtype I demonstrated the mildest motor and non-motor symptoms, best patient-reported outcomes, consistent with shortest disease duration. In contrast, Subtype III, which we have defined as a severe subtype, had worse non-motor symptoms, and reported worse motor experiences of daily living than others consistent with the longest disease duration, while Subtype II had intermediate scores on most symptoms. An interesting finding emerges from Subtype II when examining MDS-UPDRS II and MDS-UPDRS III scores, and although the MDS-UPDRS II and MDS-UPDRS III score is different for all 3 groups, the trends do not match. For MDS-UPDRS II, the patient-reported motor experiences, subtype III has a more severe phenotype compared to Subtype II. However, direct assessment of motor signs, the MDS-UPDRS III score, shows that Subtype II has a more “severe” motor phenotype in both the *on* and *off* medication states. Moreover, Subtype II does not have a change in score between the *on* and *off* medication states, in contrast to Subtypes I and III, and Subtype II also has the lowest difference of the three Subtypes between the *on* and *off* medication states. We consider this may be at least in part due to the presence of a greater degree of tremor that is poorly medication-responsive in Subtype II. This poor response could also be partly due to a lower dosage of medication for patients for Subtype II. Nonetheless, our results strongly suggest that we have identified a subgroup, Subtype II, with intermediate scores on the majority of symptom items but with medication-resistant tremor, and possibly other medication-resistant motor features.

Significant work has been done previously to identify motor subtypes in PD patients, most notably subdividing PD into TD, PIGD, and indeterminate classes. To explore the relationship between these established phenotypic subtypes and our Subtypes I-III in the present study, we examined associations in both “on” and “off” medication states. The PIGD score is highest and the TD score is the lowest in Subtype III compared with Subtypes I and II, consistent with previous studies that demonstrated that a higher PIGD score is associated with older patients who have had PD for a longer period of time^26^. Specific non-motor features, including RBD and depression are also highly represented in Subtype III. Overall our findings indicate that patients of Subtype III suffered from both worse patient-reported motor and non-motor symptoms than Subtypes I and II. However, there is a disconnect between directly observed motor scores on examination (MDS-UPDRS III) and patient-reported motor experiences of daily living (MDS-UPDRS II), suggesting that differences between groups do not simply reflect the global extent of PD severity. Moreover, examination of the motor scores also gives a unique insight to how patients respond to therapy, by comparing phenotypic subtypes based upon MDS-UPDRS III scores in the “off medication” versus “on medication” states. Individuals in Subtype II had a minimal response of motor symptom score in response to medication, and the minimal response of the tremor subscore, in contrast to Subtypes I and III. We also examined the response of the identified subtypes to medication treatment. Individuals within Subtype III were more likely to shift motor phenotypes in response to PD medication.

The biomarker measurements, CSF alpha-synuclein, CSF amyloid-Beta, CSF pTau and total Tau, and plasma alpha-synuclein did not associate with specific subtypes to the level of statistical significance. There is a question of whether any association could have been confounded by treatment, and although biospecimens were collected in the *off* state, they were only taken approximately 12 h after the last dose^5^. Moreover, biospecimen levels are stable over 6 months to a year while on medication and previous work has shown that there are little changes in these biomarkers while on medication^27^, which may be related to the lack of significant changes in this analysis. However, when analyzing DEEP SEQ mass-spectrometry data from the BioFIND study, we did identify several proteins that were present at different levels for subtype III compared to the other two subtypes. Interestingly, some of the protein biomarkers that we have identified fit with previous literature. FTL, seen to be significantly lower in the most severe subtype, has been shown to be downregulated in late-stage PD dopamine neurons^28^, and iron metabolism changes have been shown to be associated with PD^29^. Both APOA4 and C9 were also increased in the most severe phenotype. APOA4 upregulation has been associated with PD, and C9 has been shown to be higher expressed in PD patients as compared to Alzheimer’s Disease patients^30,31^. Further studies can be done to study these markers in more detail to understand the mechanistic changes that occur in patients with more severe disease. In addition, we found no difference in genetic risk scores between groups.

This study is an initial attempt to comprehensively integrate heterogeneous clinical study data, including motor and non-motor, for the identification of PD subtypes. Our approach has demonstrated the strong potentials of the identification of meaningful PD subtypes based upon cross-sectional data in the BioFIND cohort. However, there are still some limitations in the current study. Firstly, the subtyping approach taken in this study is data-driven without the utilization of any clinical domain knowledge. Appropriately combining data-driven approach with clinical domain knowledge will help the data-driven model to capture pathology and etiology, and hence enhance the subtyping results. Secondly, the study only focuses on the BioFIND population, which has limited PD samples (103 patients). One key next step would be to see if this method for subtyping generalizes well for other cohorts of moderate-to-late stage PD.

In conclusion, a comprehensive subtyping method which is based on similarity fusion was used in our study. Two kernels were first calculated to model PD patient similarity interns of motor and non-motor manifestations. Then the kernels were integrated and fed to clustering analysis. Within the BioFIND population, three clinical subtypes of PDs were detected. The identified subtypes show distinct characteristics: one subtype shows more severe motor and non-motor deficits than others; one shows mild symptoms; and one shows moderate symptoms. We also compared the newly identified subtypes with the traditional motor and sleep disorder subtypes and reveals interesting relationships.

## Methods

### Study population

BioFIND (http://biofind.loni.usc.edu) is an observational, multi-center, cross-sectional study of moderate-to-advanced PD participants evaluated with standardized clinical and biospecimen acquisition protocols^5^. Enrolled PD participants met the United Kingdom PD Society Brain Bank (UKPDBB) clinical diagnostic criteria, modified to require all three classic motor signs of PD (tremor, bradykinesia, and rigidity) instead of just two signs. The duration of the disease in the enrolled subjects was 5–18 years since the onset of motor symptoms, and those who had undergone deep brain stimulation or ablative surgeries for PD were excluded. BioFIND inclusion was limited to 50–75 years of age at the onset of the disease (age 55 to 93 at the time of enrollment). BioFIND contains two different patient states in the database for MDS-UPDRS III and IV scores (IV scores were incomplete for visit 2 and not used). The baseline or visit 1 assessment is performed while the patient is on medication and visit 2 represents a period of time when the patient is off medication. The institutional review board of BioFIND approved the study protocol. Written informed consent was obtained from each study participant.

### Data collection

We included a wide range of data available from the BioFIND study for analysis as follows:

- Demographics: age, sex, race, family history and education year.
- Motor manifestations: Movement Disorder Society Unified PD rating scale (MDS-UPDRS) Part II (motor experiences of daily living) and Part III (motor examination), Schwab & England Activities of Daily Living Scale, total tremor score, postural instability-gait difficulty (PIGD) score, and tremor/PIGD phenotype^8^. For other motor features lacking specific rating scales in this cohort (dyskinesias and dystonia), we used the single items from MDS-UPDRS Part IV (motor complications).
- Non-motor manifestations: MDS-UPDRS Part I (non-motor experiences of daily living), Montreal Cognitive Assessment (MoCA), REM sleep behavior disorder (RBD) Questionnaire, and RBD phenotype^9,32^. For other non-motor features lacking specific rating scales for measurement in this cohort (hallucination, apathy, pain and fatigue), we used the single items from MDS-UPDRS Part I.
- Medications: Levodopa Equivalent Dose. The medication was taken from the BioFIND concomitant medication log, and was derived based on the frequency of drug taken, the amount of drug per dosage, and the type of drug administered^33^.
- Biospecimen: CSF (Cerebrospinal fluid) Aβ_1-42_, total Tau (t-tau) and phosphorylated Tau (p-tau) were performed by Luminex xMAP technology using INNO-BIA Alz Bio3 kits—Fujirebio Diagnostic INC. The concentration of alpha-synuclein in CSF and Plasma samples collected for BioFind were analyzed using an ELISA assay available commercially from BioLegend^5^. All data was directly extracted from the BioFIND database.
- Genetic information: The genetic risk score was calculated previously, and described below^34^. The effects of the risk loci were reported in BioFIND as a single genetic risk score, calculated by summing the risk allele counts for the 28 common risk loci identified in the most recent large-scale meta-analysis of PD genome-wide association study (GWAS) data, as well as including the p.N370S risk variant in the GBA gene, and the p.G2019S mutation in the LRRK2 gene^5,35^. The allele counts per variant were normalized based on the log odds ratios, and effect estimates for each variant were taken from previous literature, with information for the two added alleles for GBA and LRRK2 coming from the PDgene database and 23andMe.
- Mass-spectrometry: DEEP SEQ mass-spectrometry was performed at Dana-Farber Cancer Institute and is described in depth in the BioFIND database. To analyze this data, we removed any proteins that had missing data and removed all proteins that had very small changes between datapoints (standard deviation of less than 0.3), leaving 261 proteins of interest.

Specifically for clustering, the motor features used were the MDS-UPDRS II score, the MDS-UPDRS III score at baseline on medication (chosen to correspond to the medication state for all other measures), and the Schwab and England Activities of Daily Living Score, and the non-motor features used were the MDS-UPDRS I score, the Montreal Cognitive Assessment (MoCA) score, and REM sleep behavior disorder (RBD) Questionnaire score.

### Subtyping methods overview

**Fig. 2:**
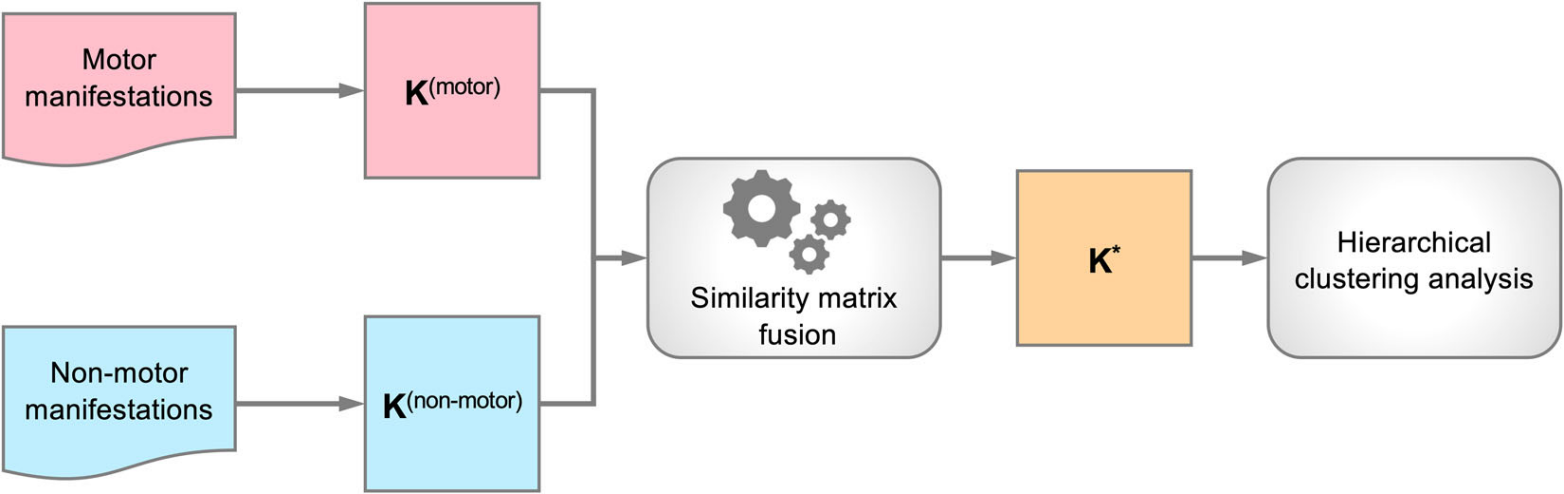
Overall workflow of PD subtyping. Two patient similarity matrices (**K**^(motor)^ and **K**^(non-motor)^) were first derived using motor and non-motor manifestation data, respectively. Then our similarity matrix fusion algorithm was performed to produce integrated similarity matrix (i.e., K*). Finally, hierarchical clustering analysis was applied to K* to identify PD subtypes.

Figure 2 illustrates the overall workflow of our subtyping technique. Two patient similarity matrices were first derived based on motor and non-motor features respectively. Then our similarity matrix fusion algorithm was performed to produce an integrated similarity matrix. Finally, hierarchical agglomerative clustering analysis was performed on the integrated similarity matrix to identify PD subtypes.

### Data preparation

All data were collected from BioFIND study (http://biofind.loni.usc.edu) and is publicly available. Data extraction was performed using Python version 3.7.1 (http://www.python.org). Clinical data, including motor and non-motor, were used to derive PD subtypes. Data was cleaned such that patients with any missing information in terms of motor and non-motor data were excluded for analysis (*N* = 15). A small sample (n = 2) patients were missing drug information and were mean imputed. All feature values were normalized using z-score using the python package sci-kit learn.

### Patient similarity matrices calculation and fusion

For a specific type of data, i.e., motor or non-motor, we derived a *N* × *N* patient similarity matrix **K** (*N* is the total number of patients) and apply a gaussian kernel to each matrix. In this way, we derived two similarity matrices **K**^(motor)^ and **K**^(non-motor)^, using motor and non-motor manifestation data, respectively (see Fig. 2). In order to appropriately combine motor and non-motor symptoms to identify subtypes, a multiple similarity matrix fusion technique, the Multiple Bregmanian Bi-Stochastication (MBBS) algorithm^25^, was performed based on **K**^(motor)^ and **K**^(non-motor)^. Specifically, MBBS is able to learn an optimal linear convex combination of multiple similarity matrices to derive an integrated one, **K**^+^, on which the data cluster structure can be best revealed. The optimal set of combination coefficients can be interpreted as the importance of each type of data for measuring patient-wise similarity (more detailed information in the Supplementary Notes).

### Cluster analysis

The integrated patient similarity matrix was converted to a distance/dissimilarity matrix, by simply taking 1−K^*^. Agglomerative hierarchical clustering was performed on the distance matrix to identify PD subgroups (scipy)^36^. In order to determine the optimal number of cluster as well as hyperparameters of similarity matrix fusion, the Silhouette index (sci-kit learn)^37^ was used to estimate clustering performance (see more details in the Supplementary Notes). According to the Silhouette index, the optimal cluster number is three, where the integrated similarity matrix ${{{\mathbf{K}}}}^ \ast = 0.6994 \times {{{\mathbf{K}}}}^{\left( {{{{\mathrm{motor}}}}} \right)} + 0.3006 \times {{{\mathbf{K}}}}^{\left( {{{{\mathrm{non}}}} - {{{\mathrm{motor}}}}} \right)}$.

### Cluster stability

To evaluate stability and robustness of identified subtypes, sensitivity analyses were conducted. We performed both patient-based sensitivity as well as data type-based sensitivity. More specifically for the patient-based analysis, the original patient population was subset at random 10 times so that 10% of the population was removed. Each time the fusion and clustering algorithm with hyperparameter tuning was performed using 100 different parameter tunes per subset. The Kuhn–Munrkres algorithm was used to match the new clusters generated from the subset of data with the corresponding cluster assignments for each patient from the original full dataset^38^. We evaluated the overlap between the clusters produced by the sensitivity analysis and that by the primary analysis. For the data type-based analysis, we determined how the addition of a different type of data would affect our analysis. We added a biomarker based matrix consisting of Aβ from CSF, α-synuclein from CSF, α-synuclein from plasma, p-Tau from CSF and Tau from CSF, to form a third separate similarity matrix. All data was z-scored normalized using sci-kit-learn. The optimization process was identical to the baseline method, but the optimization metric changed. Instead of optimizing silhouette score, rand index (sci-kit learn), which is a measure of how similar two sets of clusters are, was optimized for 50 rounds. Using this process, we obtained hyperparameters such that the coefficients for each matrix were ${{{\mathbf{K}}}}^ \ast = 0.4337 \times {{{\mathbf{K}}}}^{\left( {{{{\mathrm{motor}}}}} \right)} + 0.3600 \times {{{\mathbf{K}}}}^{\left( {{{{\mathrm{non}}}} - {{{\mathrm{motor}}}}} \right)} + 0.2063 \times {{{\mathbf{K}}}}^{\left( {{{{\mathrm{biomarker}}}}} \right)}$.

### Statistical analysis

We further assessed the differences in demographics, clinical characteristics (motor and non-motor), biomarkers and genetic information (genetic risk score) among the identified subtypes/clusters. Continuous/discrete data were described using means (standard deviations), while categorical data were described using frequency counts. Several different statistical tests were performed depending on the feature value distributions.

To analyze continuous variables, first, a QQ plot and a Levene Test were performed to look at the normality of the data as well as the homogeneity of variance respectively. If the null hypothesis for the Levene Test was rejected, an ANOVA was performed with white adjustment to account for the unequal variances. In addition, a two-sided pairwise t-test with unequal variance assumptions were done with Benjamini-Hochberg correction for a post-hoc analysis. If the null hypothesis for the Levene test could not be rejected, and the QQ plot showed a normal distribution, an analysis of variance (ANOVA) test was performed, and a Tukey’s HSD test was used for post-hoc analysis if the ANOVA p-value < 0.05. If the null hypothesis could not be rejected, and the QQ plot did not show a normal distribution, a Kruskal-Wallis test with Wilcoxon rank sum test with Benjamini-Hochberg correction. Fisher’s Exact Tests were performed for categorical data, with Benjamini-Hochberg post-hoc tests performed for multiple hypothesis correction for each cluster comparison. For data in which an ANOVA test could be performed, analysis of covariance (ANCOVA) was performed adjusting age and disease duration at baseline. The nominal p-values were also corrected using Bejamini-Hochberg FDR correction.

For mass-spectrometry analysis, an ANOVA test was performed for each protein, and FDR correction was performed to identify proteins with a corrected p-value of less than 0.05. Using the statsmodels package in python, multiple comparison testing with Benjamini–Hochberg post-hoc analysis was used to identify the subtypes that were statistically significantly different.

### Reporting summary

Further information on research design is available in the Nature Research Reporting Summary linked to this article.

## Supplementary information

Supplementary Information

Reporting Summary

## Data Availability

The BioFIND dataset used in this study can be accessed at https://biofind.loni.usc.edu/. The cleaned datasets analyzed in this study are available from the corresponding author on reasonable request.

## Code availability

The code used for subtyping can be found at https://github.com/mbbrendel/MBBS-Subtyping.
