# Supplementary material for: Comprehensive subtyping of Parkinson’s disease patients with similarity fusion: a case study with BioFIND data
Source: NPJ Parkinsons Dis. 2021 Sep 17;7:83. doi: 10.1038/s41531-021-00228-0 (PMC8448859; doi:10.1038/s41531-021-00228-0)
Supplement: Supplementary file 1 — Supplementary Information [file 41531_2021_228_MOESM1_ESM.pdf]

Supplementary File

Title: Comprehensive Subtyping of Parkinson's Disease Patients with Similarity Fusion: A Case Study with BioFIND Data

Authors: Matthew Brendel, Chang Su, Yu Hou, Claire Henchcliffe, Fei Wang

Supplementary Notes

A. Similarity matrix generation and fusion

For a specific type of data, i.e., motor or non-motor, we derived a $N \times N$ patient similarity matrix $\mathbf{K}$ (N is the total number of patients) whose (i, j)-th element $\mathbf{K}_{ij}$ the similarity score between patients i and j defined using a Gaussian function as follows

$$\mathbf{K}_{ij} = exp^{\left(-\frac{\rho^2(x_i, x_j)}{2\sigma_{ij}^2}\right)} \tag{1}$$

Here, x_i and x_j were feature vectors of patients i and j, respectively; $\rho(x_i, x_j)$ was the Euclidean distance between i and j; and the scaling parameter was defined as

$$\sigma_{ij} = \mu \cdot \frac{mean_{u \in N_i}\big(\rho(x_i, u)\big) + mean_{v \in N_j}\left(\rho\big(x_j, v\big)\right) + \rho\big(x_i, x_j\big)}{3} \tag{2}$$

where, μ is a pre-defined parameter and N_i is the k nearest neighbors of i[5-8]. In this way, we derived two similarity matrices $\mathbf{K}^{(1)}$ and $\mathbf{K}^{(2)}$, using motor and non-motor manifestation data, respectively (see Figure 1).

In order to appropriately combine motor and non-motor symptoms to identify subtypes, a multiple similarity matrix fusion technique developed by us, the Multiple Bregmanian Bi-Stochastication (MBBS) algorithm[1], was performed based on $\mathbf{K}^{(\text{motor})}$ and $\mathbf{K}^{(\text{non-motor})}$. Specifically, MBBS is able to learn an optimal linear convex combination of multiple similarity matrices to derive an integrated one, $\mathbf{K}^*$, on which the data cluster structure can be best revealed. The optimal set of combination coefficients can be interpreted as the importance of each type of data for measuring patient-wise similarity.

B. Parameter determination

In this analysis, there were 4 different hyperparameters that needed to be tuned, cluster number s, parameter μ and k in Gaussian kernel function, and regularization coefficient λ of Multiple Bregmanian Bi-Stochastication (MBBS) algorithm[1]. Silhouette index[2] was used to estimate clustering performance. The Hyperopt tool[3] was used to tune the hyperparameters by optimizing

the Silhouette index. One limitation was that the minimum number of patients per cluster had to be greater than 10, to prevent all patients from being grouped into only one cluster. The Tree-structured Parzen Estimator (TPE) algorithm was used for tuning, as this has been shown to improved performance over random search and grid search[4]. We used 200 rounds of optimization for the hyperopt tool.

Supplementary Figures

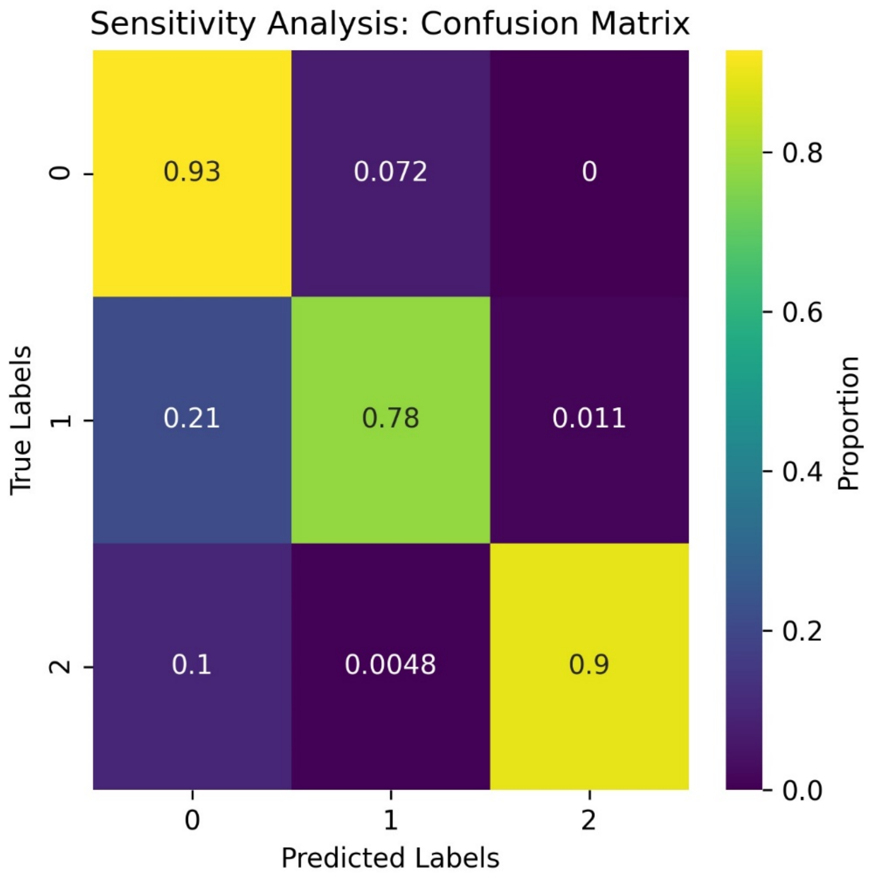

Supplementary Figure 1: Cluster stability analysis. Clustering was performed 10 times, and the figure above shows the aggregate predictions from all 10 (Average per subset with 95% confidence interval: 0.891 (0.763-1.0). On the y axis is true labels and the x axis is predicted labels. All data is normalized to the total number of true labels per cluster (normalized by row).

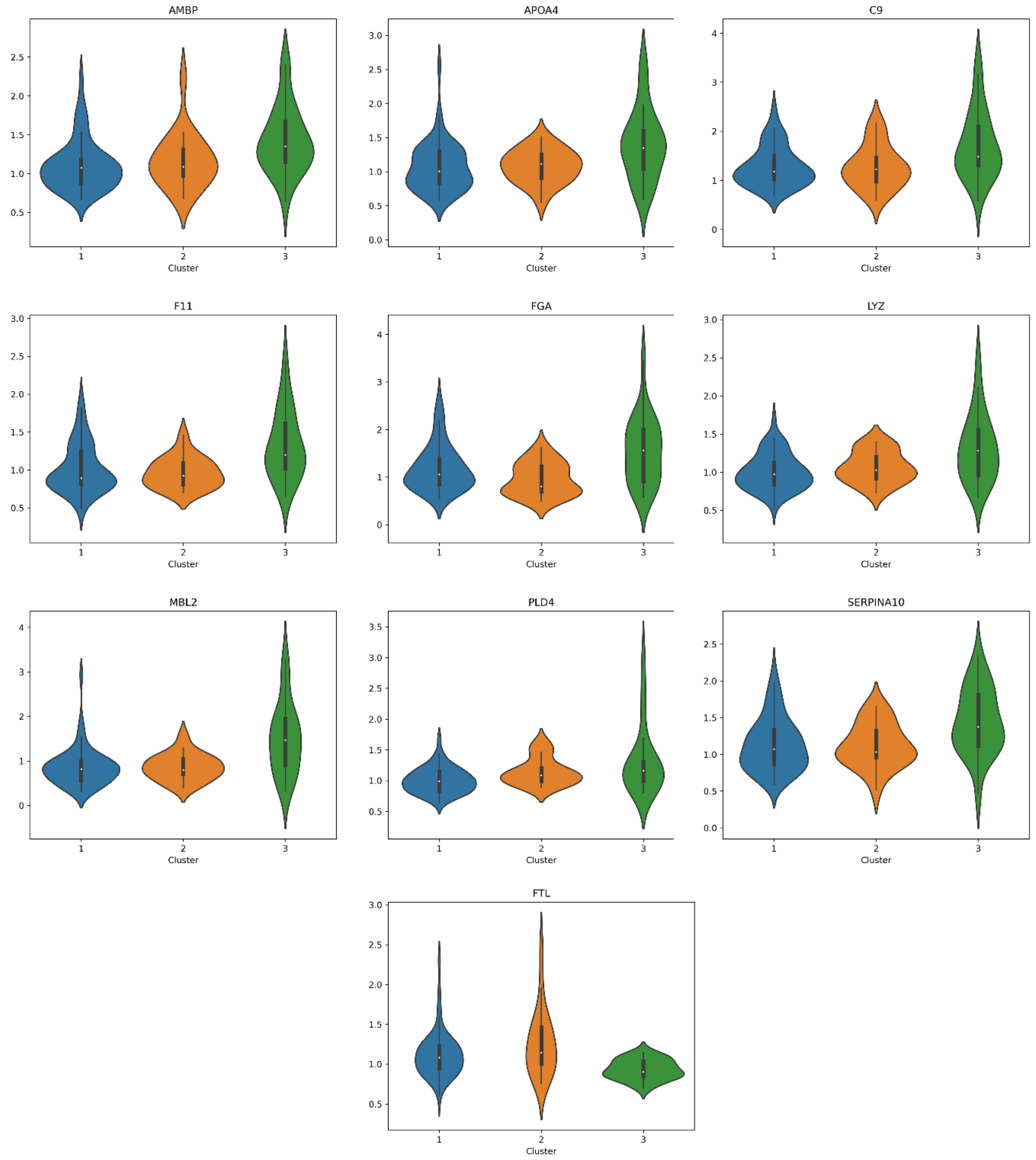

Supplementary Figure 2: Violin plots showing the distribution of mass-spectrometry results split by clusters. The y axis represents a relative quantitative evaluation of protein levels in the CSF and x axis represents the clusters. The violin plots show a kernel density estimate of the feature distribution. The white dot within each plot represents the median, the edges of the box represent the interquartile and the line represents 1.5 times the interquartile range.

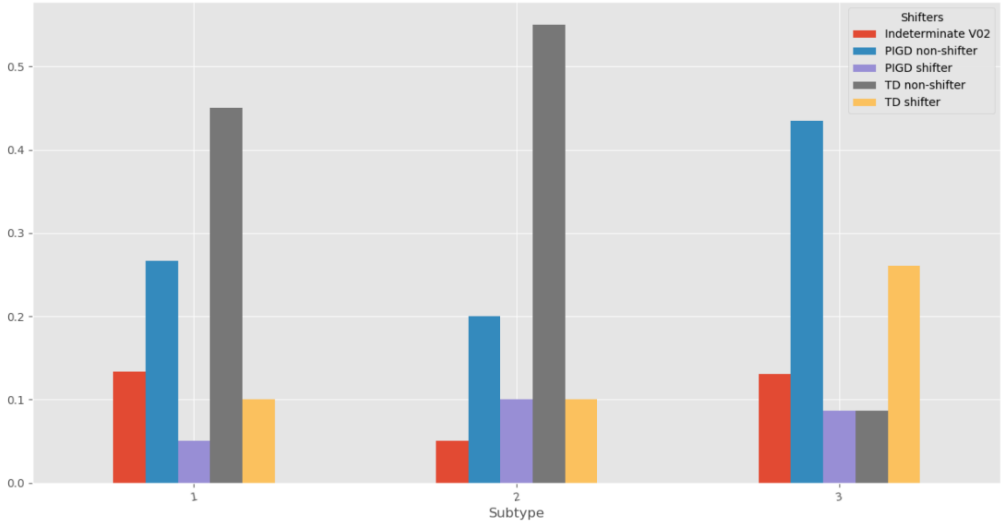

Supplementary Figure 3: Bar plot showing the distribution of motor subtypes within the three clusters, and how they change between on and off state. Data represents frequency of each class within a specific subtype.

References

1. Wang, F., Li, P., König, A. C. & Wan, M. Improving clustering by learning a bi-stochastic data similarity matrix. *Knowl Inf Syst* **32**, 351–382 (2012).

2. Rousseeuw, P. J. Silhouettes: A graphical aid to the interpretation and validation of cluster analysis. *Journal of Computational and Applied Mathematics* **20**, 53–65 (1987).

3. Bergstra, J., Yamins, D. & Cox, D. D. Making a Science of Model Search: Hyperparameter Optimization in Hundreds of Dimensions for Vision Architectures. 9.

4. Bergstra, J. S., Bardenet, R., Bengio, Y. & Kégl, B. Algorithms for Hyper-Parameter Optimization. 9.

5. Wang, B. *et al.* Similarity network fusion for aggregating data types on a genomic scale. *Nat Methods* **11**, 333–337 (2014).

6. Wang, B., Zhu, J., Pierson, E., Ramazzotti, D. & Batzoglou, S. Visualization and analysis of single-cell RNA-seq data by kernel-based similarity learning. *Nat Methods* **14**, 414–416 (2017).

7. Zhou, D., Bousquet, O., Lal, T. N., Weston, J. & Schölkopf, B. Learning with Local and Global Consistency. 8.
8. Zhu, X., Ghahramani, Z. & Lafferty, J. Semi-Supervised Learning Using Gaussian Fields and Harmonic Functions. 8.
