# Supplementary material for: Comprehensive subtyping of Parkinson’s disease patients with similarity fusion: a case study with BioFIND data
Source: NPJ Parkinsons Dis. 2021 Sep 17;7:83. doi: 10.1038/s41531-021-00228-0 (PMC8448859; doi:10.1038/s41531-021-00228-0)
Supplement: Supplementary file 2 — Reporting Summary [file 41531_2021_228_MOESM2_ESM.pdf]

nature portfolio

Corresponding author(s): Fei Wang

Last updated by author(s): Aug 5, 2021

Reporting Summary

Nature Portfolio wishes to improve the reproducibility of the work that we publish. This form provides structure for consistency and transparency in reporting. For further information on Nature Portfolio policies, see our Editorial Policies and the Editorial Policy Checklist.

Statistics

For all statistical analyses, confirm that the following items are present in the figure legend, table legend, main text, or Methods section.

n/a	Confirmed	
☐	☒	The exact sample size (*n*) for each experimental group/condition, given as a discrete number and unit of measurement
☐	☒	A statement on whether measurements were taken from distinct samples or whether the same sample was measured repeatedly
☐	☒	The statistical test(s) used AND whether they are one- or two-sided<br>*Only common tests should be described solely by name; describe more complex techniques in the Methods section.*
☐	☒	A description of all covariates tested
☐	☒	A description of any assumptions or corrections, such as tests of normality and adjustment for multiple comparisons
☐	☒	A full description of the statistical parameters including central tendency (e.g. means) or other basic estimates (e.g. regression coefficient) AND variation (e.g. standard deviation) or associated estimates of uncertainty (e.g. confidence intervals)
☒	☐	For null hypothesis testing, the test statistic (e.g. *F*, *t*, *r*) with confidence intervals, effect sizes, degrees of freedom and *P* value noted<br>*Give P values as exact values whenever suitable.*
☒	☐	For Bayesian analysis, information on the choice of priors and Markov chain Monte Carlo settings
☒	☐	For hierarchical and complex designs, identification of the appropriate level for tests and full reporting of outcomes
☒	☐	Estimates of effect sizes (e.g. Cohen's *d*, Pearson's *r*), indicating how they were calculated

Our web collection on statistics for biologists contains articles on many of the points above.

Software and code

Policy information about availability of computer code

Data collection	Data collection was performed by BioFIND.
Data analysis	The code used for subtyping can be found at https://github.com/mbbrendel/MBBS-Subtyping. Implementation of our work, including data preparation, clustering model for subtyping, and statistical tests, were based on Python 3.7. The involved Python packages included Pandas 1.2.1, NumPy 1.20.1, scikit-learn 0.23.2, statsmodels 0.11.1, seaborn 0.9.0, matplotlib 3.0.3.

For manuscripts utilizing custom algorithms or software that are central to the research but not yet described in published literature, software must be made available to editors and reviewers. We strongly encourage code deposition in a community repository (e.g. GitHub). See the Nature Portfolio guidelines for submitting code & software for further information.

Data

Policy information about availability of data

All manuscripts must include a data availability statement. This statement should provide the following information, where applicable:

- Accession codes, unique identifiers, or web links for publicly available datasets
- A description of any restrictions on data availability
- For clinical datasets or third party data, please ensure that the statement adheres to our policy

The BioFIND dataset used in this study can be accessed at https://biofind.loni.usc.edu/. The cleaned datasets analyzed in this study are available from the corresponding author on reasonable request.

March 2021

Field-specific reporting

Please select the one below that is the best fit for your research. If you are not sure, read the appropriate sections before making your selection.

☒ Life sciences ☐ Behavioural & social sciences ☐ Ecological, evolutionary & environmental sciences

For a reference copy of the document with all sections, see nature.com/documents/nr-reporting-summary-flat.pdf

Life sciences study design

All studies must disclose on these points even when the disclosure is negative.

Sample size	103 PD participants from the BioFIND study were used for analysis
Data exclusions	Patients have any missing values in clinical variables for clustering were excluded for analysis.
Replication	To evaluate stability and robustness of identified subtypes, sensitivity analyses were conducted. We performed both patient-based sensitivity as well as data type-based sensitivity. More specifically for the patient-based analysis, the original patient population was subset at random 10 times so that 10 percent of the population was removed. Each time the fusion and clustering algorithm with hyperparameter tuning was performed using 100 different parameter tunes per subset. The Kuhn-Munrkres algorithm was used to match the new clusters generated from the subset of data with the corresponding cluster assignments for each patient from the original full dataset. We evaluated the overlap between the clusters produced by the sensitivity analysis and that by the primary analysis. For the data type-based analysis, we determined how the addition of a different type of data would affect our analysis. We added a biomarker based matrix consisting of Aβ from CSF, α-synuclein from CSF, α-synuclein from plasma, p-Tau from CSF and Tau from CSF, to form a third separate similarity matrix. All data was z-scored normalized using scikit-learn. The optimization process was identical to the baseline method, but the optimization metric changed. Instead of optimizing silhouette score, rand index (sci-kit learn), which is a measure of how similar two sets of clusters are, was optimized for 50 rounds.
Randomization	We derived subtypes from all patients included for analysis.
Blinding	This is not relevant to our study because the research team was not involved with the data collection process.

Reporting for specific materials, systems and methods

We require information from authors about some types of materials, experimental systems and methods used in many studies. Here, indicate whether each material, system or method listed is relevant to your study. If you are not sure if a list item applies to your research, read the appropriate section before selecting a response.

Materials & experimental systems

n/a	Involved in the study	
☒	☐	Antibodies
☒	☐	Eukaryotic cell lines
☒	☐	Palaeontology and archaeology
☒	☐	Animals and other organisms
☒	☐	Human research participants
☐	☒	Clinical data
☒	☐	Dual use research of concern

Methods

n/a	Involved in the study	
☒	☐	ChIP-seq
☒	☐	Flow cytometry
☒	☐	MRI-based neuroimaging

Clinical data

Policy information about clinical studies

All manuscripts should comply with the ICMJE guidelines for publication of clinical research and a completed CONSORT checklist must be included with all submissions.

Clinical trial registration	NA
Study protocol	NA
Data collection	103 PD participants from the BioFIND study were used for analysis. We collected demographics, motor and non-motor clinical data, and biospecimen data of the patients for analysis.
Outcomes	NA
